# *Bacillus subtilis*: a universal cell factory for industry, agriculture, biomaterials and medicine

**DOI:** 10.1186/s12934-020-01436-8

**Published:** 2020-09-03

**Authors:** Yuan Su, Chuan Liu, Huan Fang, Dawei Zhang

**Affiliations:** 1 College of Biotechnology, Tianjin University of Science and Technology, Tianjin, 300457 China; 2 Tianjin Institute of Industrial Biotechnology, Chinese Academy of Sciences, Tianjin, 300308 China; 3 Key Laboratory of Systems Microbial Biotechnology, Chinese Academy of Sciences, Tianjin, 300308 China; 4 University of Chinese Academy of Sciences, Beijing, 100049 China

**Keywords:** *Bacillus subtilis*, Genetic manipulation, Protein expression, Biochemicals, Enzymes, Antimicrobials, Biofilms

## Abstract

Due to its clear inherited backgrounds as well as simple and diverse genetic manipulation systems, *Bacillus subtilis* is the key Gram-positive model bacterium for studies on physiology and metabolism. Furthermore, due to its highly efficient protein secretion system and adaptable metabolism, it has been widely used as a cell factory for microbial production of chemicals, enzymes, and antimicrobial materials for industry, agriculture, and medicine. In this mini-review, we first summarize the basic genetic manipulation tools and expression systems for this bacterium, including traditional methods and novel engineering systems. Secondly, we briefly introduce its applications in the production of chemicals and enzymes, and summarize its advantages, mainly focusing on some noteworthy products and recent progress in the engineering of *B. subtilis*. Finally, this review also covers applications such as microbial additives and antimicrobials, as well as biofilm systems and spore formation. We hope to provide an overview for novice researchers in this area, offering them a better understanding of *B. subtilis* and its applications.

## Introduction

*Bacillus subtilis* is an aerobic, Gram-positive soil bacterium, which has been widely used for the production of heterologous proteins [1]. It secretes numerous enzymes to degrade a variety of substrates, enabling the bacterium to survive in a continuously changing environment. This species and some of its close relatives have excellent protein secretion ability, making them important hosts for the production of medicinal proteins and industrial enzymes. For these reasons, it has been widely used to produce heterologous proteins. Moreover, it has excellent physiological characteristics and highly adaptable metabolism, which makes it easy to cultivate on cheap substrates. Accordingly, *B. subtilis* grows fast and the fermentation cycle is shorter, usually, around 48 h, while the fermentation cycle of *Saccharomyces cerevisiae* is around 180 h [2, 3]. Furthermore, excellent expression systems with good genetic stability are available for this organism, and it has no strong codon preference. Different from *Escherichia coli*, *B. subtilis* has a single cell membrane, which facilitates protein secretion, simplifies downstream processing, and reduces the process costs. Finally, this species is generally recognized as safe (GRAS) [4, 5].

Over the decades of research, many different tools for genetic modification of *B. subtilis* have been developed, including the classical counter-selection marker strategies and recently developed clustered regularly interspaced short palindromic repeats (CRISPR)-Cas9/Cpf1 based tool box. Its diverse protein secretion systems, as well as the recently developed artificial promoter and ribosome binding site (RBS) libraries are also helpful in the production of extracellular enzymes. The newly discovered expression cassette integration (MEXI) method based on the mariner transposon can produce knock-in mutants with higher levels of intracellular GFP and extracellular AprE expression than the commonly used *amyE* integration method [6], thus improving the production of heterologous proteins. In addition to being an excellent host in bioreactors, *B. subtilis* is an ideal multifunctional probiotic, with great potential for preventing the growth of pathogenic bacteria and enhancing nutrient assimilation [7]. *B. subtilis* is also commonly used as an industrial cell factory, for the production of vitamins, inositol, acetoin, hyaluronan, and other chemicals. Its clear inherited backgrounds and well-developed gene manipulation tools enabled the reconstruction of its cellular metabolism, and the availability of public knockout collections makes them attractive as metabolic engineering hosts [8]. Yang Gu et al. redesigned the central carbon and redox metabolism of *B. subtilis* with a new “push-pull promote” approach, through which they manipulated the central carbon metabolism, eliminated the metabolic overflows, and achieved high production of N-acetylglucosamine (GlcNAc) [9]. In agriculture, studies have shown that adding an appropriate amount of *B. subtilis* can significantly improve the humus and carbon content of compost, thus improving soil quality and promoting crop growth [10]. *B. subtilis* can also form complex biofilms, which can be used as living biological materials for the production of many functional biomaterials, such as surface growth factors, antibiotics, lysozyme, and antimicrobial peptides for medical materials.

**Fig. 1:**
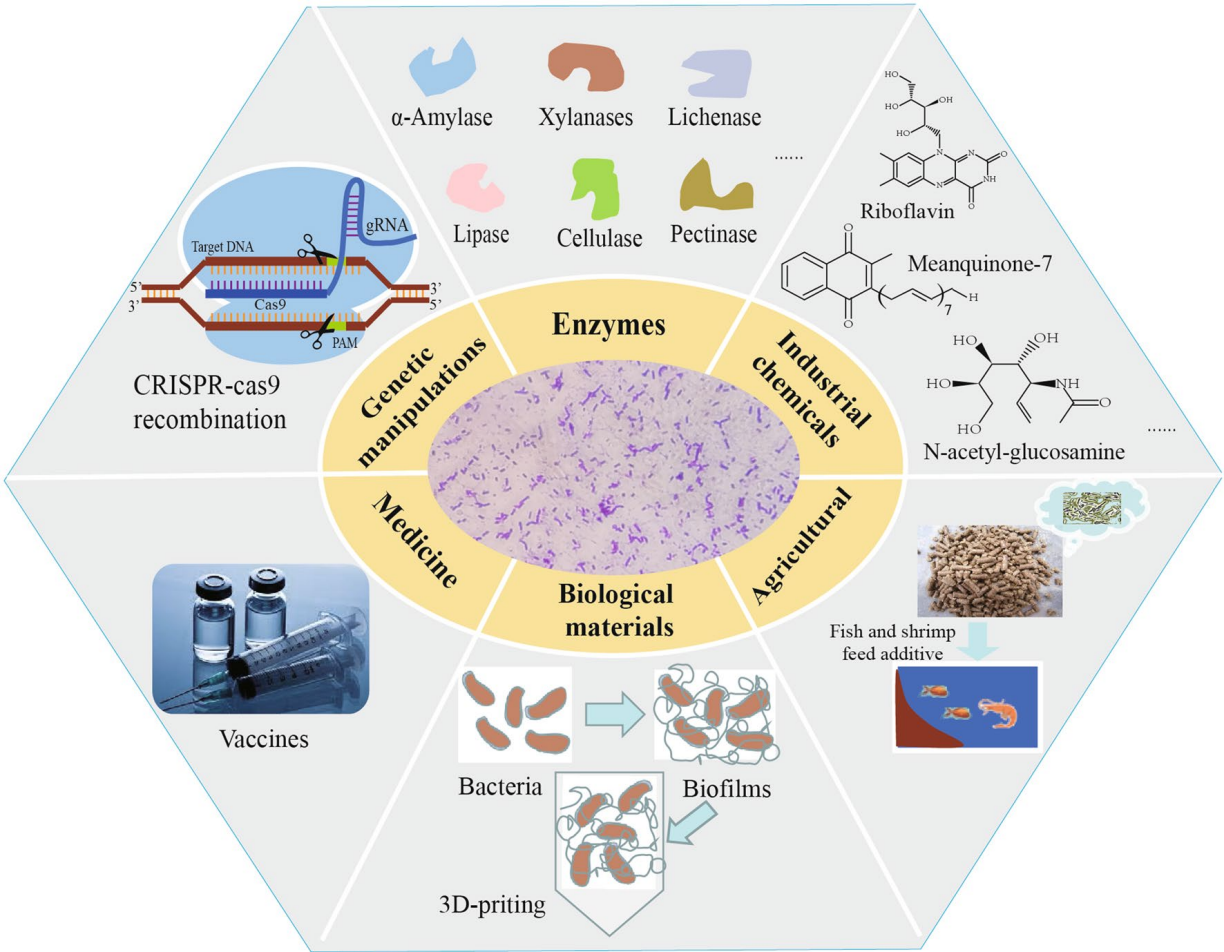
Application of *B. subtilis* for genetic engineering, production of industrial chemicals or enzymes, agriculture, medicine and biomaterials. The CRISPR/cas9 tool has been widely used in the genetic engineering of *B. subtilis*. The bacterium can be used to produce various industrial enzymes, such as α-amylase, xylanase, lichenase, lipase, cellulase, or pectinase. It can also be used to produce various chemicals, such as riboflavin, menaquinone-7, inositol, or N-acetylglucosamine. In agriculture, it can be used as a feed additive. Biofilms of *B. subtilis* can be used as a biomaterial in 3D printing. In medicine, *B. subtilis* can be used to produce vaccines

In this paper, we reviewed recent progress in the metabolic engineering and protein expression systems, as well as industrial, agricultural, and biomaterial applications of *B. subtilis*. Finally, we analyzed the factors that hinder the further application of this strain and discussed the reasons. This review provides a reference for researchers who want to gain a general understanding of *B. subtilis* and its various applications (Fig. 1).

## Genetic manipulation of ***Bacillus subtilis***

As *B. subtilis* was selected as a model bacterium, simple and efficient genetic tools have been developed in the past decades. Classical genome modification relies on the insertion of a selectable marker, usually an antibiotic resistance gene, into the chromosome of the target strain [11]. The most commonly used scarless genetic manipulations systems for *B. subtilis* rely on counter-selectable markers (CSM) [12], while other methods include site-specific recombination systems (SSR) [13], and the recently developed CRISPR-Cas9 system [14].

**Fig. 2:**
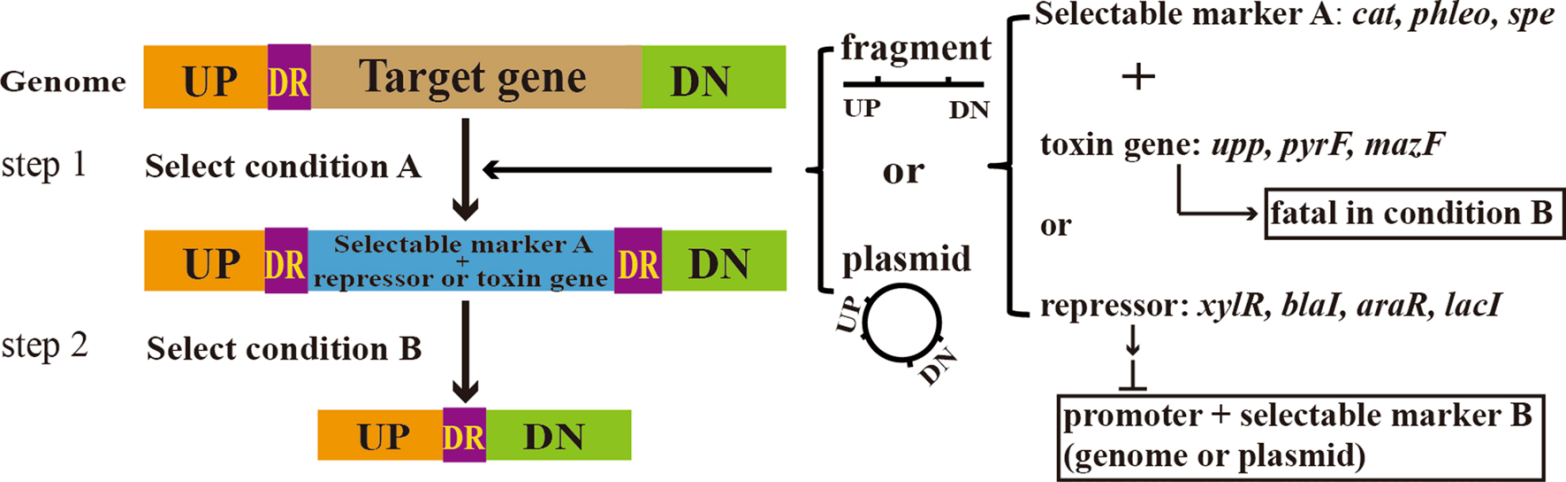
Schematic overview of genome editing methods based on counter-selectable markers. Left: genome editing (gene knockout as an example) with two integration steps. Step 1, an exogenous artificial DNA (plasmid or fragment) with up- and downstream homologous sequences is integrated into the genome, replacing the target gene. The recombinant clone can be selected under condition A. Step 2, under the selection condition B, the clone obtained in step 1 deleted the selectable marker and repressor/toxin gene through a self-recombination with the DR (direct repeats). Right: Composition of the selectable elements, the selectable marker A, toxin gene/repressor, and up- and downstream homologous sequences of the target gene can be constructed as fragments or on a plasmid. Examples of selectable markers A include: *cat* (chloramphenicol), *phleo* (phleomycin), or *spe* (spectinomycin). Examples of toxic genes include: *upp*, *pyrF*, or *mazF*. Examples of repressor genes include: *xylR*, *blaI*, *araR*, or *lacI*. The repressor can inhibit the expression of the selectable marker B, which can be integrated into the genome or a plasmid

CSM are often used for the markerless construction of engineered strains and have been used to construct *Bacillus* cell factories for various industrial applications. Selectable markers can generally be divided into positive and negative selection markers, whereby the former are most commonly antibiotic-resistance markers. In this classical approach, antibiotic-resistant strains are selected on appropriate agar plates (Fig. 2). In addition to the genomically integrated markers, Jeong et al. constructed a synthetic gene circuit consisting of a plasmid-based selection system, in which the P_xyl_-*lacI* and neomycin resistant gene (*neo*) are integrated into the genome, while a P_spac_-chloramphenicol (*cat*) resistant cassette and *xylR* gene are on the plasmid. In the first recombination, P_xyl_-*lacI* and *neo* are integrated into the genome as a selectable marker. When xylose is added to the medium, the *lacI* gene is expressed then the chloramphenicol resistant gene is repressed. Consequently, the cell will survive only when the P_xyl_-*lacI* and *neo* are deleted through a second round of recombination. Finally, the plasmid can be removed after several rounds of culture without chloramphenicol [15]. This is a highly efficient method for genome engineering in *B. subtilis*, and it avoids the introduction of a selectable marker into the genome or the tightly controlled expression of a toxic gene. Other counter-selectable markers commonly used in *B. subtilis* include *upp*, *blaI*, *araR*, and *hewI* [11]. Fabret et al. used the *upp* gene, which encodes uracil phosphoribosyltransferase as a counter-selection marker to achieve the transmission of unlabeled point mutations, in-frame deletions and large numbers of deletions on the chromosome [16]. Brans et al. developed another method to knock out a single gene and introduce a new gene by combining the use of *blaI*, an antibiotic resistance gene, which encodes a repressor of the *Bacillus licheniformis* BlaP β-lactamase, with a conditional lysine-auxotrophic *B. subtilis* strain [17]. However, CSM-based strategies require host pre-modification and have a low success rate due to the leaky expression of the CSM.

Site-specific recombination (SSR) systems are powerful tools for precise excision of DNA fragments. These systems, such as *FLP*/*FRT* [18] and *Cre*/*loxP* [13], have much higher recombination efficiency than the endogenous recombination systems, making them an ideal tool for many genetic manipulations. By combining a mutated *Cre*/*lox* system with the long segment fusion PCR method [19], Yan et al. developed a rapid and accurate *B. subtilis* genome engineering tool that allows operations such as targeted gene inactivation, long-fragment deletion, and in-frame deletion of target genes [13].

In recent years, the application of the CRISPR-related (Cas) system in *B. subtilis* has further enriched the gene editing toolbox. The CRISPR locus is first transcribed into a precursor CRISPR ribonucleic acid (pre-crRNA), which is then cut into small RNA units under the action of Cas protein or endonuclease. These small RNA units are mature crRNAs that contain spacer sequences and partial repeat sequences. Maturation of the crRNAs of type II CRISPR/Cas systems requires not only the participation of Cas9 and RNase, but also the guidance of a tracrRNA [20]. Mature crRNAs and tracrRNA form double-stranded RNA structures through complementary base pairing. The resulting duplexes bind Cas9 protein to form a targeted cutting complex, specifically cutting foreign sequences to achieve the goal of identifying and eliminating invading foreign genes such as plasmids and viruses [21–23]. At present, there are three kinds of CRISPR/Cas9-based genome editing strategies widely used in *B. subtilis*. (1) The single-plasmid based system, in which Cas9, a single guide RNA (gRNA), donor DNA, and other elements are assembled into the same carrier skeleton, wherein Cas9 protein and gRNA are respectively expressed from inducible or strong constitutive promoters; (2) The two-plasmid-based system is more flexible than the single plasmid system. In this system, Cas9, gRNA, and donor DNA are assembled on two different plasmids, which are respectively used to produce Cas9 protein and deliver the gRNA transcription module and donor DNA template; (3) The chromosomally integrated system is more stable and effective than the first two systems, but it requires the use of engineered strains. The Cas9 was integrated into the genome, and then araE/R initiation subsystem was used to construct a multi-gRNA delivery vector [22]. Furthermore, this CRISPR-Cas9 toolkit was extended to CRISPR interference (CRISPRi) for transcriptional-level regulation [21]. The CRISPRi system is composed of a deactivated Cas9 (dCas9) protein and gRNA, enabling the targeting of dCsa9 to any target gene on the genome under the guidance of gRNA to inhibit its transcription without inducing a double-strand break, which can be applied to gene repression in metabolic engineering [24]. So et al. developed a CRISPR-derived genome engineering technique to efficiently generate large genomic deletions in *B. subtilis* without the introduction of counter-selectable markers such as antibiotic-resistance genes, which had previously limited the application of *B. subtilis* in food engineering [25]. This method has wide applicability for various types of site-directed mutagenesis in *B. subtilis* [24, 26, 27]. However, CRISPR/Cas9 has low efficiency in multi-gene editing. Liu et al. recently developed a CRISPR/Cas9n-mediated genome editing system, using Cas9n to exchange the natural Cas9 of existing constructs for *B. subtilis* and for iterative editing of the genome [28]. This system is more effective than CRISPR/Cas9 in various types of gene modification and shows higher efficiency for large genomic deletions or multiplex gene editing. In terms of multi-gene editing and regulation, the newly developed CRISPR/Cpf1 system is the most powerful tool in *B. subtilis*. Compared with CRISPR/Cas9, CRISPR/Cpf1 has higher targeting specificity and can be used for gene editing in human cells, plant cells and many bacteria, while also offering a higher efficiency of multiplex gene editing [29]. In fact, the system provides up to 100% efficiency of double in-frame knockouts, enables the introduction of multiple point mutations (up to six) with 100% efficiency, and can be used to simultaneously activate and/or inhibit multiple genes [27].

## Gene expression in ***Bacillus subtilis***

To enhance and appropriately adjust gene expression levels in *B. subtilis*, it is essential to study the promoters that regulate transcription levels [30]. Inducible and constitutive promoters are usually applied for the expression of heterologous genes in *B. subtilis* [31]. In addition to the promoters, the protein expression level is also influenced by the strength of the ribosome binding site, while plasmids with different copy numbers also provide choices for adjusting the level of gene expression [31]. Different genes have specific expression features, and their expression must be adjusted to a level appropriate for a specific metabolic pathway, which necessitates the use of promoters with different strengths for engineering purposes [32]. Researchers have broadened the scope of target gene transcription levels by constructing promoter libraries. In a recent study, Liu et al. constructed a synthetic promoter library with an intensity gradient by analyzing microarray transcriptome data of *B. subtilis* 168, which can be used for extensive fine-tuning of genetic pathways in *B. subtilis*, facilitating strain engineering and synthetic biology [33]. To enable the efficient and accurate co-expression of multiple genes in metabolic networks, a recent review discussed the construction promoter libraries by site-directed mutagenesis [34], such as error-prone PCR, saturation mutagenesis and directional design. RBS sequences can be used to fine-tune gene expression at the translational level. When inducible promoters are used, different ribosomal binding sites can be used to fine-tune the dynamic range of gene expression. In addition, a proteolysis tag can be used to control the degradation rate of a protein at the post-translational level [35]. A synthetic gene expression toolbox consisting of promoter libraries, RBS libraries, and different proteolytic tags can realize gene regulation with a dynamic range of 5 orders of magnitude [36]. Other elements other than the promoter and RBS are also used to adjust gene expression in *B. subtilis*. Tian et al. conducted and statistical analyzed 96 rationally selected N-terminal coding sequences (NCSs) from *B. subtilis*, which influenced gene expression at the translation level. They found that NCS substitution is more efficient and convenient than promoter substitution for gene expression improvement [37]. Naseri et al. discussed the construction of complex libraries and combinatorial optimization strategies. However, the examples shown in their review indicate that *E.coli* and *S. cerevisiae* are used as host more often than *B. subtilis* [38]. Therefore, future studies should focus on developing new tools for this important Gram-positive model bacterium.

## Protein secretion systems of ***Bacillus subtilis***

**Fig. 3:**
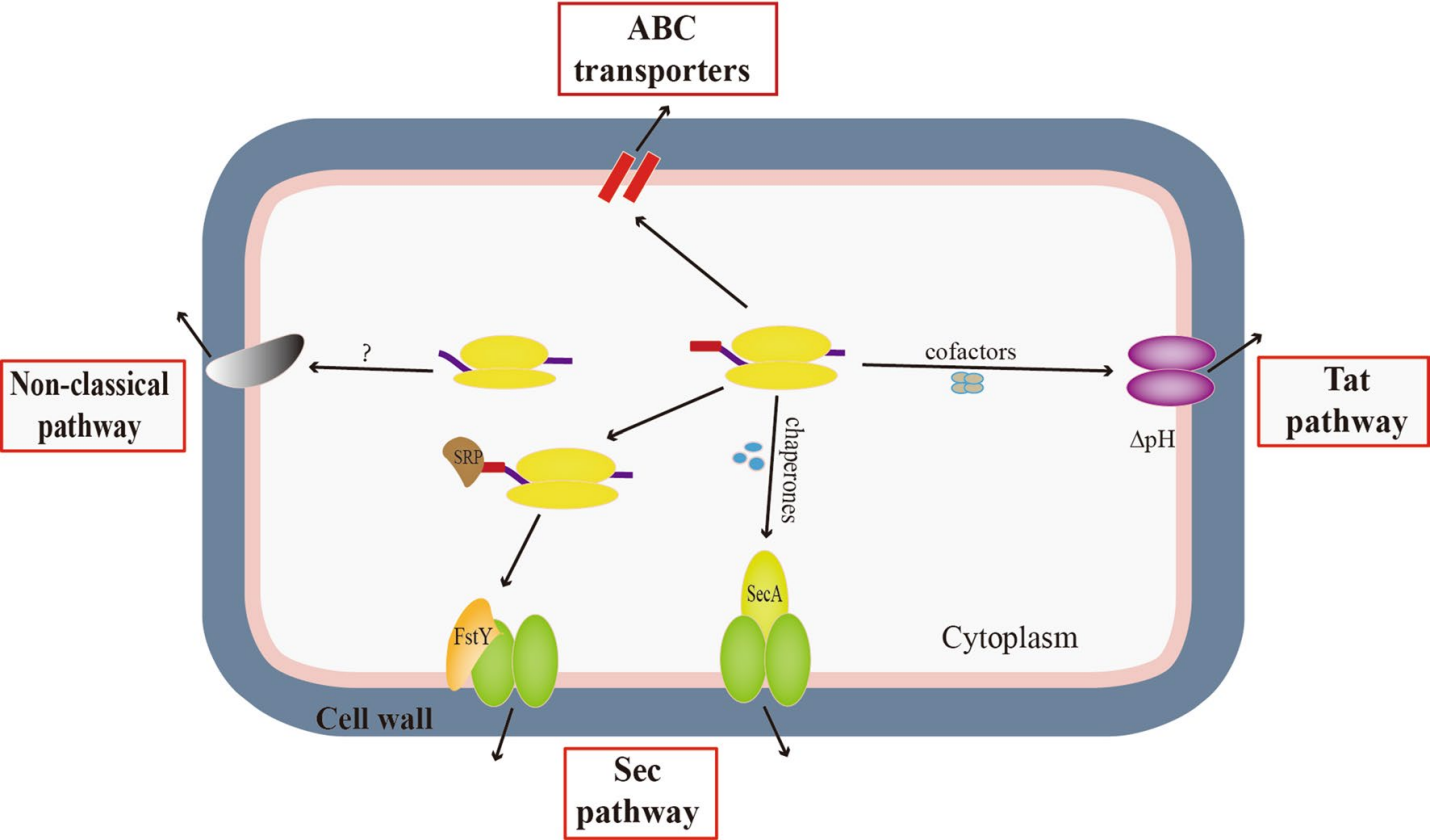
Schematic diagram of protein secretion pathways in *B. subtilis*. The mechanism of the non-classical secretion pathway is not clear

*Bacillus subtilis* has a strong capacity for protein expression and secretion, which has led to its wide use in the production of industrial enzyme preparations. In addition to the abundant promoters and plasmid expression systems described above, *B. subtilis* also has an efficient protein secretion system to meet the needs of the secretion of various proteins. There are three classical protein secretion pathways in *B. subtilis*, the general protein secretion pathway (Sec), the twin-arginine translocation pathway (Tat), and the ATP-binding cassette (ABC) transporters [39] (Fig. 3). The Sec pathway is the main transport channel, which can transport a large number of exported proteins. The essential elements of the Sec pathway are the signal recognition particle (SRP), Sec translocase, type I signal peptidase, and chaperones. After the synthesis of the precursor protein, there are two routes through the Sec pathway. In the first one, the signal peptide is recognized by the signal recognition particle (SRP) with the help of a cytoplasmic chaperone, and then transferred to the membrane to bind with FstY, the receptor protein of the SRP, and transported to the channel of the Sec translocase complex (translocation channel). The second route maintains the precursor protein’s translocation ability by preventing its complete folding using intracellular chaperones, and the it is then transfer to the Sec translocase complex. Next, the N-terminal signal peptide sequence is cut off by signal peptidases. Finally, the translocating protein is folded in the extracellular space with the aid of extracellular chaperones [30, 40]. In contrast to the Sec pathway, which relies on unfolded substrates, the Tat pathway transports tightly folded proteins that contain a conserved twin-arginine motif in the signal peptide sequences. Before translocation, the precursors fold in the cytoplasm with the help of cofactors, and are then excreted through the Tat protein complex (Tat translocase) utilizing energy from the pH gradient across the cytoplasmic membrane. After translocation, the type I signal peptidase processes the signal peptide, and finally, the folded mature proteins are secreted out of the cell. ATP-binding cassette (ABC) transporters contain two transmembrane domains (TMD) that define substrate binding sites, and two soluble nucleotide binding domains (NBD) that act as motor domains [30, 39]. They are relatively specific for their substrates and can export or import various molecules (ions, amino acids, peptides, antibiotics, polysaccharides, proteins, etc.) [39].

In addition to the three classical protein secretion pathways mentioned above, researchers also found that many non-classical secretion pathways are used to secrete non-classical proteins lacking any known signal peptides or secretion motifs [41]. Wang et al. used four typical non-classical secretory proteins as signals to direct the export of the nucleoskeletal-like protein (Nsp) from *B. subtilis* cells. Two of them were able to guide the export of alkaline phosphatase (PhoA), and one of them was able to guide the secretion of the thermostable reporter protein β-galactosidase (BgaB) [42]. Chen et al. used d-psicose 3-epimerase (RDPE) to directly secrete two of five foreign proteins from other bacteria. The fusion proteins not only retained the corresponding enzymatic or biological activities, but also had the activity of RDPE [43] (Fig. 3).

## Industrial application of chemicals produced by ***Bacillus subtilis***

The industrial application of *B. subtilis* has developed rapidly in the last decades, and it has become the major microbial cell factory for many industrial products [44, 45], including enzymes [46], heterologous proteins [31], antibiotics [47], vitamins [48], and amino acids [26]. Chemicals produced by *B. subtilis* also play an important role in various fields, such as food, feed, cosmetics, chemicals, and pharmaceuticals. Here, we will focus on vitamins and other chemicals produced by *B. subtilis*, among which the vitamin B_2_, vitamin K, *scyllo*-inositol, hyaluronic acid, and N-acetylglucosamine will be discussed in detail. Table 1 also lists some other representative chemicals produced by *B. subtilis*.

**Table 1:** Representative chemicals produced by *B. subtilis*

Products	Strains	Characteristics	Titer	References
Riboflavin	*B. subtilis* 125	The deregulation of the *rib* operon and purine de novo synthesis pathway	4232 ± 34.42 mg/L	[50]
Menaquinone-7	*B. subtilis* 20-QT	Co-overexpression of *tatAD-CD* and *qcrA-C*	410 mg/L	[60]
*Scyllo*-Inositol	*B. subtilis* KU303	Deletion of *iolABCDEF*, *iolHIJ*, *iolX* and *iolR*, combined with the expression of *IolG*, *IolW*, *IolT* and *PntAB*	27.6 g/L	[61]
Hyaluronic acid	*B. subtilis* E168TH	Expression of *hasA*, *tuaD*, *gtaB*, *glmU*, *glmM*, *glmS*, and *H6LHyal*	19.38 g/ L	[64]
N-acetylglucosamine	*B. subtilis* 168 BNDR122	Deletion of *nagP*, *gamP*, *gamA*, *gamR*, *nagA*, *nagB*, *ldh*, *alsRSD*, *pta*, *ackA*, *glcK, pckA, pyk*, *lacA* and *amyE*, combined with the expression of *GNAI*	131.6 g/L	[67]
Amorphadiene	*B. subtilis* 1A1	Overexpression of *dxs*, *idi* and *ads*	20 mg/L	[96]
Poly-γ-glutamic acid	*B. subtilis* ZJU − 7	Optimizing culture conditions	101.1 g/L	[97]
Acetoin	*B. subtilis* CGMCC 13,141	Deletion of *araR*, *bdhA* and *acoA*	83.7 g/L	[98]
Shikimate	*B. subtilis* 168 CLC6-PYKA	Deletion of *pykA* and *aroI*	4.67 g/L	[99]
2,3-Butanediol	*B. subtilis* F9	Deletion of *upp*, *acoA*, *bdhA*, *pta*, and *ldh*, combined with the expression of *alsS*, *alsD*, *budC*, and *udhA*	103.7 g/L	[100]
Isobutanol	*B. subtilis* UL08	Deletion of *alsS*, *ldh*, *pdhC* and *pgi*, combined with the expression of *zwf* and *udhA*	6.12 g/L	[101]
Chondroitin	*B. subtilis* E168H	Expression of *kfoC*-*kfoA* and *kfiC*-*kfiA*, combined with the upregulation of *tuaD*	5.22 g /L	[102]
Heparosan	*B. subtilis* E168H	Expression of *kfoC*-*kfoA* and *kfiC*-*kfiA*, combined with the upregulation of *tuaD*	5.82 g/ L	[102]

## Vitamins as high-value products

In a recent review, microbial cell factories for the production of B vitamins were described in detail [48]. *B. subtilis* can be used to produce vitamins B_1_, B_2_, B_5_, B_6_, and B_7_, and many production strains were constructed by metabolic engineering or screened and selected from mutant libraries [48]. Vitamin B_2_ is one of the most successful microbial fermentation products on an industrial scale [49]. Also known as riboflavin (RF), Vitamin B_2_ is the precursor of flavin mononucleotide and flavin adenine dinucleotide [50], and it is widely used for its antioxidant, immunity enhancing, anticancer, as well as food and feed enhancing effects [51]. Metabolic engineering strategies based on the riboflavin metabolic pathway of *B. subtilis* are usually based on optimization of the central carbon metabolism, overexpression and deregulation of the RF synthesis and purine biosynthesis pathways, as well as blocking the synthesis of by-products [52]. The precursor supply in the RF biosynthesis pathway can be enhanced by redirecting the carbon flux through the PPP (pentose phosphate pathway) from the EMP (Embden-Meyerhof-Parnas) pathway, and increasing the expression of purine biosynthesis genes (*pur* operon), thus increasing GTP production [53, 54]. The yield of riboflavin in fed-batch fermentation using *B. subtilis* reached up to 826.52 mg/L [54]. The excellent RF productivity of *B. subtilis* can also be attributed to its excellent productivity of chemicals sharing the same precursors, such as ribose, purine nucleoside, and folic acid. In addition, the yield of RF can be increased by improving the host characteristics. Recent studies found that the introduction of heat shock proteins from thermophilic bacteria can improve the heat resistance and osmotic tolerance of *B. subtilis*, enabling it to ferment at a higher temperature, thereby shortening the fermentation time and improving the RF titer [55].

In addition to B vitamins, *B. subtilis* can also produce menaquinone-7 (MK-7), a member of the valuable vitamin K_2_ family, which can promote blood coagulation and osteogenic ability, and is also known for other nutraceutical and pharmacological properties. In the fermentation process of MK-7, glycerol, glucose, sucrose, or starch are used as carbon sources, and yeast extract, peptone, sodium nitrate, or soybean peptone as nitrogen sources [56]. Using ethanol to extract MK-7 directly from the cells after fermentation produced a MK-7 yield of 1.47 mg/g [57]. Wu and Ahn optimized the medium components via a three-step response surface methodology (RSM) approach, and the yield of vitamin K increased to 71.95 ± 1.00 mg/L [58]. Yang et al. found that the expression of *menA*, *dxs*, *dxr*, *yacM*, *yacN* and *glpD* constitutes bottlenecks for MK-7 production. Knocking out *dhbB* can promote the production of MK-7, and it was further improved by adopting high-density fermentation technology [59]. In addition, MK-7 is an important component of the microbial membrane, where it plays an important role in the process of electron transport and oxidative phosphorylation. Cui et al. regulated the membrane composition and electron transport by co-expression of the cell membrane protein *tatAD-CD* and menaquinol-cytochrome lyase *qcrA-C*, which increased the titer of MK-7 to 410 mg/L in shake flasks [60].

## *Scyllo*-inositol (SI)

*Scyllo*-inositol (SI), a stereoisomer of inositol, is being investigated as a potential therapeutic agent for Alzheimer’s disease. However, this compound is far less abundant than its analog *myo*-inositol (MI), which is the most abundant inositol stereoisomer in nature and can be obtained from rice bran. Consequently, many studies have investigated strategies to convert MI into SI using microorganisms. Tanaka et al. constructed a strain with modified inositol metabolism by deleting all genes related to inositol metabolism, and overexpressing the key enzymes, IolG and IolW, in *B. subtilis*, resulting in a cell factory that can convert MI into SI with increased efficiency [61]. Through the genetic modification of inositol metabolism and phytase secretion pathway of *B. subtilis*, the signal peptide of *B. subtilis* was optimized, the main MI transporter, IolT, was overexpressed, and the substrate absorption was improved. At the same time, the *pnt*AB gene of *E. coli* was introduced to improve the NADPH production, which improves the activity of inositol dehydrogenase, IolW. These modifications successfully increased the conversion efficiency of MI to 30 g/L/48 h [62].

## Hyaluronic acid (HA)

Hyaluronic acid (HA) is a high-value glycosaminoglycan, which is widely used in the biomedical, pharmaceutical, cosmetic, and food industries. Notably, HA preparations with different molecular weight show different effects [63]. Peng Jin et al. downregulated the glycolysis pathway by co-expressing identified committed genes (*tuaD*, *gtaB*, *glmU*, *glmM*, and *glmS*). They used the ribosome binding site engineering strategy to regulate the translational level of hyaluronidase and optimized the HA synthesis pathway, which led to the specific production of low-molecular-weight HA [64]. Li et al. recently reported an engineered strain of *B. subtilis* that can produce HA with different molecular weights and titers at different temperatures. They found that when the biomass increased, the molecular weight of the produced HA decreased while the titer increased [63].

## N-acetylglucosamine (GlcNAc)

In addition to naturally occurring products, scientists are also introducing new pathways into *B. subtilis* to produce new chemicals. N-acetylglucosamine is an acetylated amine derivative of glucose, which plays an important role in the maintenance and repair of cartilage and joint tissue function. Liu et al. divided the GlcNAc biosynthesis network into a glycolysis module, a GlcNAc biosynthesis module, and a peptidoglycan biosynthesis module. By applying modular pathway engineering, they increased the production of GlcNAc in *B. subtilis* from 1.85 to 31.65 g/L [65]. Then, by knocking out acetolactate synthase (AlsS) and acetolactate decarboxylase (AlsD), the formation of the neutral byproduct acetoin was reduced, and the carbon flux from fructose-6-phosphate toward the GlcNAc synthesis pathway was increased. Consequently, the titer and yield of GlcNAc increased to 48.9 g/L and 0.32 g/g glucose, respectively [66]. The catabolism of GlcNAc in *B. subtilis* is regulated by GlcN6P. Recently, Liu et al. built a coupled ADC system consisting of a GlcN6P-responsive biosensor and CRISPRi. Using this system, a genetic feedback circuit was constructed to fine-tune the metabolic flow toward GlcNAc synthesis and competing modules, which increased the titer of GlcNAc in a 15-L fed-batch bioreactor to 131.6 g/L [67].

## Enzymes produced by ***Bacillus subtilis***

Due to its rapid growth on inexpensive substrates, strong protein secretion ability, non-pathogenicity, and favorable downstream processing, *B. subtilis* has become an ideal expression host for the production of various industrial enzymes. According to incomplete statistics, enzymes produced using *B. subtilis* account for 50% of the total enzyme market [44]. Many enzymes have been successfully expressed in *B. subtilis*, including amylases, xylanases, lichenase, β-galactosidase [68], cellulases [69], alkaline serine proteases [42], and many others. These enzymes play important roles in the food, feed, detergent, textile, leather, paper, and pharmaceutical industries [46]. Due to its GRAS status, proteases from *B. subtilis* can be used in various food applications, such as soybean hydrolysate preparation, meat tenderization, casein hydrolysate preparation, milk coagulation, and food waste treatment [70]. Here, we will introduce illustrative examples of engineered *B. subtilis* factories for the production of enzymes such as amylases, xylanases, and lichenases.

## Alpha-amylase

Alpha-amylase (EC 3.2.1.1) catalyzes the cleavage of α-1,4-glucosidic bonds, releasing glucose from starch. It is widely used in the textile and paper industries, and *B. subtilis* is a major host for the production of heterologous α-amylases [71]. Ma et al. used atmospheric and room temperature plasma mutagenesis followed by a novel screening method, and combined with a fermentation optimization strategy, significantly improved the yield of alkaline amylase in *B. subtilis* 168 [72]. Other studies focused on regulating protein transport and transcription levels by integrating signal peptides and promoter engineering [73]. PrsA is an effective folding catalyst for proteins expressed in *B. subtilis*. Overexpression of native PrsA from *B. subtilis* can improve the yield of amylase, but for heterologous amylase, co-expressing the cognate *prsA* gene had a better effect. QuesadaGanuza et al. developed a new recombinant PrsA variant, which not only increased the yield of amylase in *B. subtilis*, but also relieved the secretion pressure of the strain [74]. By optimizing the signal peptide and overexpression partner, followed by error-prone PCR and high throughput screening technology to screen improved mutants, the α-amylase activity was improved to 9201.1 U/mL [75].

## Xylanases

Xylanases (EC. 3.2.1.8) are enzymes that catalyze the hydrolysis of β-1,4 glycosidic linkages of xylans, releasing oligosaccharides and disaccharides containing reducing sugars and xylose [76]. They have significant application value in biotechnology and can be used to modify lignocellulosic materials. Xylanases are used in animal feed manufacturing, the paper and textile industries, and biofuel production. Commercial xylanases are mainly produced in *B. subtilis*. Sanchez-Alponti et al. created and characterized single mutants individually replacing five residues in the mesophilic xylanase of *B. subtilis* with homologous residues from thermophilic enzymes. When the five mutants were combined in a random combinatorial library, a double mutant with improved specific activity and thermal stability was obtained [77]. Yardimci and Cekmecelioglu used Box–Behnken response surface methodology to optimize xylanase production in a co-culture of *B. subtilis* and *Kluyveromyces marxianus*, which improved the xylanase yield 4.4-fold (reached 49.5 IU/mL) compared to the initial un-optimized single culture [76].

## Lichenase

Lichenase (EC. 3.2.1.73) is a mixed linked β-glucan (MLG) endo-hydrolase found in both microorganisms and plants, which has become a focus of studies on the feasibility of biofuel production [78]. However, due to its poor thermal stability, it is not suitable for biocatalytic biomass conversion. Wang et al. used SpyTag/SpyCatcher-mediated cyclization and non-chromatographic ITC purification to obtain cyclo-lichenase with good heat resistance, which is superior to linear lichenase [78]. In addition, in terms of the catalytic activity of the enzyme, studies have shown that the assembly of subunits induced by the collagen triple helix domain and essential oligomers, such as comp and foldon, can cause target enzyme trimerization, thus improving the activity of lichenase [79].

## Application of ***Bacillus subtilis*** in agriculture, biomaterials and medicine

Because it is a non-pathogenic probiotic, *B. subtilis* is often used as a microbial additive to improve intestinal function in animals. It was found promote animal growth and prevent diseases [80]. It can be manufactured in the form of endospores, which then enter the intestinal tract of animals and quickly reactivate to secrete highly active proteases, as lipases and amylases in the upper intestinal tract, which is helpful to degrade complex carbohydrates in plant feed. Furthermore, *B. subtilis* can produce polypeptides that have an antagonistic effect against intestinal pathogens, effectively improving the digestibility of feed. Additionally, it can be used in water bioremediation and prevent diseases in aquaculture organisms such as shrimp and fish [7]. Because *B. subtilis* is an aerobic bacterium, it contributes to the anaerobic environment by consuming oxygen in the intestines, which in turn promotes the reproduction of dominant bacteria in the intestine, maintaining the ecological balance of intestines.

*B. subtilis* can secrete a variety of low molecular weight antimicrobial peptides and bacteriocins, such as surfactin [81], bacilysin [82], and subtilin [83], which have potential value in biomedical engineering, food and agriculture [84]. Antimicrobial peptides from *B. subtilis* are promising therapeutic tools, because of their broad activity and rapid killing activity against a variety of pathogens. With the increasing problems of microbial resistance due to the non-rational use of conventional antibiotics, antimicrobial peptides will play a more significant role in the treatment of bacterial infections [85]. Antimicrobial peptides also have the advantages of safety and environmental friendliness. They are widely used as feed additives in agriculture and animal husbandry to enhance animal fiber digestion and intestinal health. *B. subtilis* can improve the balance of intestinal flora and has the potential to improve intestinal health and food absorption efficiency. Studies have shown that adding *B. subtilis* spores to dairy cattle feed can improve milk and protein production [86]. Furthermore, adding *B. subtilis* to the diet of laying hens can improve their performance as well as the shell quality of eggs produced by aged laying hens [87].

Biofilms are structured communities of tightly associated cells that constitute the predominant state of bacterial growth in natural and human-made environments [88]. Biofilms can be used to produce living materials with self-healing functions, which are desirable for many products [89]. *B. subtilis* can form complex and robust biofilms and is a good model strain for studying biofilm formation. Recent studies have shown that biofilms based on the TasA amyloid protein mechanism in *B. subtilis* exhibit viscoelastic behavior of hydrogels. Studies have shown that biofilms can be accurately fabricated into various three-dimensional (3D) microstructures through 3D-printing and microencapsulation technology [90]. Compared with chemical materials, this artificial living material has metabolic activity, self-renewal ability, and programmability [89]. In addition, the ability of *B. subtilis* to form biofilms promotes the synthesis of quorum-sensing pentapeptide and nitric oxide (NO), which can delay the aging of the host [91]. Biofilm formation improves the ability of microorganisms to metabolize nutrients and produce chemicals, and can be used to improve the stability of fermentation processes. Recent studies show that a biofilm reactor can promote the extracellular secretion of MK-7 [92].

When faced with starvation, *B. subtilis* produces endospores that can survive for a long time in a state of anabiosis. And when nutrients are available, it germinate again [93]. This characteristic is conducive to the successful expression of heterologous antigens or increasing the relative activity, thermostability, pH stability, and reusability of enzymes on the surface of spores. The enormous stress resistance of endospores can be coopted to improve the stability and promote the reusability of enzymes in complex environments [94]. In addition, spore surface display technology has recently been successfully applied in the production of protein polymers, vaccines and industrial enzymes [95]. Enzymes such as lipase and chitinase have been successfully expressed on the surface of endospores [94].

### Conclusions and future perspectives

As the main model species of Gram-positive bacteria, *B. subtilis* has a broad array of mature genetic tools, promoters, and plasmid expression systems, which can be used in metabolic engineering, protein expression, and synthetic biology. It can produce chemicals, enzymes, and other industrial bio-products, but also be used as a platform for vaccine preparation or a feed additive in agriculture. Moreover, it is also an ideal model for studying biofilm formation and other physiological characteristics of attached cells. This paper reviewed the advantages of *B. subtilis* as a chassis cell from several aspects, including genetic manipulation, and heterologous gene expression, as well as its application in industry, agriculture, and medicine.

However, although *B. subtilis* has various attractive applications, it remains far less studied than its Gram-negative counterpart, *E. coli*. This remains true even in the present era with rich methodology and rapid tool development. One major bottleneck in the application of new methods attribute to the lower efficiency of plasmids construction in *B. subtilis* compared with *E. coli*. To solve this, future engineered strains of *B. subtilis* can be developed to allow direct plasmid construction. Conversely, the high recombination rate of *B. subtilis* has some advantages for the development of genome editing tools.

Our goal was to provide reads with a general understanding of the characteristics of *B. subtilis*, so one can take advantage of its features for certain applications or research purposes. Of course, as more and more scientists and engineers contribute studies on *B. subtilis*, more technologies, tools, and methods will be applied to *B. subtilis* in the future, and the potential of this bacterium for scientific and industrial applications will be enhanced further.

## Data Availability

Not applicable.

## Abbreviations

RBS: Ribosome binding site
GlcNAc: N-acetylglucosamine
CSM: Counter-selectable marker
SSR: Site-specific recombination system
CRISPR: Clustered regularly interspaced short palindromic repeats
CRISPRi: CRISPR interference
Sec: Secretion pathway
Tat: Twin-arginine translocation pathway
ABC: ATP-binding cassette
SRP: Signal recognition particle
TMD: Transmembrane domain
NBD: Nucleotide binding domain
Nsp: Nucleoskeletal-like protein
PhoA: Alkaline phosphatase
BgaB: β-Galactosidase
RDPE: d-Psicose 3-epimerase
RF: Riboflavin
PP pathway: Pentose phosphate pathway
MK-7: Menaquinone-7
RSM: Response surface methodology
SI: *scyllo*-inositol
MI: *myo*-Inositol
HA: Hyaluronic acid
AlsS: Acetolactate synthase
AlsD: Acetolactate decarboxylase
MLG: Mixed linked β-glucan
3D: Three-dimensional
